# Identification and Engineering of Aptamers for Theranostic Application in Human Health and Disorders

**DOI:** 10.3390/ijms22189661

**Published:** 2021-09-07

**Authors:** Debleena Basu, Sourabrata Chakraborty, Riddhi Pal, Tarun Kumar Sharma, Siddik Sarkar

**Affiliations:** 1 Cancer Biology and Inflammatory Disorder, Council of Scientific & Industrial Research-Indian Institute of Chemical Biology, Translational Research Unit of Excellence (IICB-TRUE), Kolkata 700091, India; debleena@csiriicb.res.in (D.B.); sourabrata@csiriicb.res.in (S.C.); riddhi@csiriicb.res.in (R.P.); 2 Aptamer Technology and Diagnostics Laboratory, Multidisciplinary Clinical and Translational Research Group, Translational Health Science and Technology Institute, Faridabad-Gurugram Expressway, Gurugram 121001, India

**Keywords:** aptamer, systematic evolution of ligands by exponential enrichment (SELEX), aptasensor, lateral flow assay (LFA), COVID-19, theranostic, single cell proteomics

## Abstract

An aptamer is a short sequence of synthetic oligonucleotides which bind to their cognate target, specifically while maintaining similar or higher sensitivity compared to an antibody. The in-vitro selection of an aptamer, applying a conjoining approach of chemistry and molecular biology, is referred as Systematic Evolution of Ligands by Exponential enrichment (SELEX). These initial products of SELEX are further modified chemically in an attempt to make them stable in biofluid, avoiding nuclease digestion and renal clearance. While the modification is incorporated, enough care should be taken to maintain its sensitivity and specificity. These modifications and several improvisations have widened the window frame of aptamer applications that are currently not only restricted to in-vitro systems, but have also been used in molecular imaging for disease pathology and treatment. In the food industry, it has been used as sensor for detection of different diseases and fungal infections. In this review, we have discussed a brief history of its journey, along with applications where its role as a therapeutic plus diagnostic (theranostic) tool has been demonstrated. We have also highlighted the potential aptamer-mediated strategies for molecular targeting of COVID-19. Finally, the review focused on its future prospective in immunotherapy, as well as in identification of novel biomarkers in stem cells and also in single cell proteomics (scProteomics) to study intra or inter-tumor heterogeneity at the protein level. Small size, chemical synthesis, low batch variation, cost effectiveness, long shelf life and low immunogenicity provide advantages to the aptamer over the antibody. These physical and chemical properties of aptamers render them as a strong biomedical tool for theranostic purposes over the existing ones. The significance of aptamers in human health was the key finding of this review.

## 1. Introduction

The concept of DNA interaction with protein can be dated back to the days of discovery of the DNA foot-printing experiment in 1978, which was primarily used in search of DNA sequences specifically interacting with ligands (proteins or small molecules) [1]. Nucleic acid–protein interactions were used to study the specificity of sequences of DNA/RNA to bind with transcription factors that regulated the expression of genes. The specificity of sequences of nucleotides is also evident in the interaction of recombinant endonuclease, ligase and polymerase [2]. Electrophoretic mobility shift assay or EMSA is another technology that uses the basis of the interaction of nucleic acid and protein [3], where the mobility of DNA is retarded in gel when bound with protein. Hence, it is notable that there is the affinity of nucleic acid sequences to bind with various macromolecules. Based on these observations, it is inevitable that, like antibodies, nucleic acid sequences have a certain degree of affinity to bind with protein, which can be harnessed to replace the antibodies in diagnostics and therapeutics applications. Thus, the era of the theranostic battle is highly anticipatable between the high affinity oligonucleotides (popularly known as aptamers) and the antibodies.

It is conceivable that if the random sequences will be incubated with macromolecules like a protein or enzyme, there will be some nucleic acids that bind with a different dissociation constant. The nucleic acid sequences that have lowest dissociation constant (K_d_), have the highest affinity and vice versa. This prompted Gold and his graduate student, Turek, to devise a technology named **S**ystematic **E**volution of **L**igands by **Ex**ponential enrichment (SELEX) in an attempt to identify the nucleic acid sequences having a high affinity against the RNA bound protein (bacteriphageT4 DNA polymerase; gp43) that is responsible for its own translational repression [4]. The alternate cycles of binding to the ligand and amplification of the bound nucleotide sequences from the initial pool of random sequences resulted in high affinity sequences. By this approach, the wild type and the most abundant variant form of the sequences that bound the ligand (e.g., gp43) with highest affinity were obtained. These high affinity oligonucleotide sequences were later termed as *aptamer* by Ellington and his group in 1992 [5]. The word ‘aptamer’ is obtained from two different words, i.e., ‘Aptus’ (Latin) and ‘Meros’ (Greek), which denote ‘fit’ and ‘part’, respectively. They can be developed against any targets, e.g., small molecules, proteins (ligands, receptors, etc.) or even the cells by combinatorial chemistry with molecular biology tools using traditional SELEX or its variants [6,7,8].

Interestingly, the synthesis of aptamers is completely done in vitro without any requirement of a biological system, like an antibody, and thus, batch to batch variation is minimized. Ease of chemical synthesis makes these molecules cost effective as well. It has a long shelf life; therefore, long storage can be possible easily [9]. Small size (10–15 KDa for aptamers vs. 150 KDa for antibodies; IgG), efficient tissue penetration and targeting, ref. [10] along with reduced immunogenicity, help in vivo applications and make it a candidate for future precision medicine [11,12].

Several reviews have already been published demonstrating the history, structural and functional analysis, modifications and different applications of aptamers, but this review is unique in that it highlights the combined theranostic approaches of aptamers in different aspects of human health and biomedical fields, as well as elucidating the future prospects of aptamers in detection of single cell proteomics and the potency of multivalent aptamers in immunotherapy. This review also helps researchers to develop an idea about the potential role of aptamers as an alternative molecular tool against COVID-19, which may accelerate bench to bedside research.

## 2. Applications

Although the aptamer was initially prepared as a replacement for an antibody, it shows immense applications in different fields. Aptamers are playing great roles in bioimaging, diagnosis and therapeutic arena [13].

### 2.1. Bioimaging

Bioimaging is the optical visualization of biological process where two different types of agents are required, one which helps in visual detection, i.e., a tracer molecule, and the other for carrying the former one to the target tissue, i.e., a carrier molecule. Due to its efficient tissue penetration property and high target specificity, the aptamer can be widely used in different types of bioimaging. 

#### 2.1.1. Magnetic Resonance Imaging

Magnetic Resonance Imaging or MRI needs powerful contrast agents which can increase or decrease relaxation time according to requirements. It may be longitudinal or transverse (T_1_ or T_2_), and the change of relaxation time helps in changing the brightness of an MRI signal, generating a three-dimensional image of the target [14]. Gadolinium compounds or Supermagnetic iron oxide nanoparticles are engineered with target specific aptamers in the aim of making a smarter contrast agent [15]. A short oligonucleotide coupled with gadolinium-tetraazacyclododecanetetraacetic acid (Gd-DOTA) has been synthesized, having complementarity to a part of anti-adenosine aptamer. When the aptamer recognizes adenosine, it releases the Gd-DOTA coupled oligonucleotide, which, in turn, lowers the molecular weight of the gadolinium compound and turns the MRI signal off due to the increase of its relaxation time or T_1_ [15]. The same principle can be implemented on a T_2_-weighted contrast agent, like Cross Linked Iron Oxide nanoparticles, called CLIOs [16].

#### 2.1.2. Positron Emission Tomography

In Positron Emission Tomography or PET imaging, the decaying property of a radioisotope by positron emission is used [17]. Radioisotope injected intravenously emits a positron, which interacts with the electron of tissues and generates two photons, directed 180° apart. These photons are captured by the PET camera and finally converted into an electrical signal [17]. Among various suitable radioisotopes, like ^12^C, ^18^F and ^15^O, Fluorine-18 (^18^F) is used mostly due to its physical properties, like a convenient half-life, facile production, low emission, etc. Being a non-invasive form of imaging, PET is highly recommended for the in vivo study of protein expression in a tissue-specific manner. A 41-nucleotide aptamer sgc8 against protein tyrosine kinase 7 (PTK7) was conjugated with ^18^F-benzylazide, with the aid of click chemistry, to address the challenge of imaging of PTK7 expression at the colon carcinoma tumor site in a noninvasive manner [18]. Radiolabeled sgc8 was injected into an HCT116 (overexpressing PTK7) mouse xenograft model, and PET images were taken post 30 min and 60 min of injection to quantify the level of the target protein, PTK7. Although this approach has been used to monitor the protein expression level in cell lines and mouse tumor models having differential expression of PTK7, it can have a high impact in future clinical applications to study or quantify multiple protein expressions in different human malignancies.

Apart from the above-mentioned methods, aptamers have also been used in Single Photon Emission Computed Tomography (SPECT), Computed Tomography (CT), Ultrasound (US) and fluorescence imaging for in vitro as well as in vivo purposes [19,20]. Broccoli, Spinach, Corn and Mango are some of the fluorescent aptamer candidates that have been studied extensively to enhance fluorescence activity and bioavailability [20].

### 2.2. Diagnostics

An aptamer, when it works as a sensor molecule in the detection of its target, is called an Aptasensor. Different types of aptasensors are available, like electrochemical aptasensor, fluorogenic aptasensor, colorimetric aptasensor, etc.

#### 2.2.1. Fluorescent Detection

**Figure 1:**
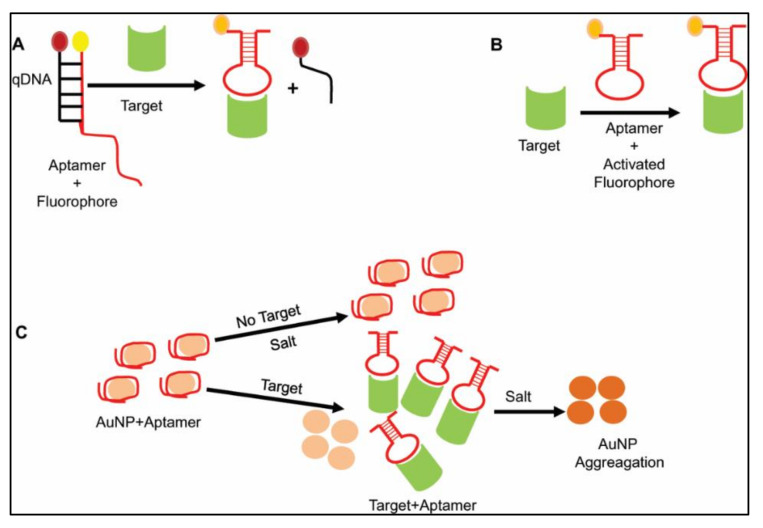
Schematic representation of aptamer-mediated detections. (**A**) Target-induced structural conversion mode: The fluorophore-tagged aptamer binds with a short oligonucleotide sequence having a quencher molecule by base pair complementarity. In the absence of a target molecule, the quencher molecule quenches fluorophore. Upon target binding, the conformational change in the aptamer helps to release qDNA and finally fluoresce. (**B**) Direct binding-based mode: The fluorophore-tagged aptamer directly binds with target. (**C**) Target-induced dissociation mode: Gold nanoparticles (AuNP) are coated onto the aptamers. Salt-mediated aggregation of AuNPs after the aptamer–target interaction finally leads to target detection by a colorimetric assay.

A complementary oligo-containing quencher molecule binds with an aptamer, which is released upon target recognition, leading to fluorescence enhancement (Figure 1A). Nowadays, in place of the traditional quencher, nanomaterials like quantum dots or QD (artificial spherical fluorescent nanocrystals having a semiconductor property), gold nanoparticles (AuNP), graphene oxide or GO (single atomic layered compound synthesized by oxidation of graphite), etc. are used for their superior quenching property. As an example, a cocaine-specific aptamer was tagged with fluorescein (fluorophore) and a DABCYL moiety (quencher) [21]. In a different study, Zhao et al. detected aflatoxin B1, for which they used a 29 mer aptamer [22]. They tagged the aptamer with FAM and used a complementary 14 mer cDNA tagged with BHQ1 as a quencher. When the target molecule was absent, the aptamer paired with cDNA by base complementarity to quench the fluorescence. Fluorescence recovery took place when the quencher DNA (qDNA) was released, upon aptamer-mediated aflatoxin recognition. This technique will be of great use for medical and food safety purposes [22]. This method is sometimes bypassed by direct conjugation of fluorophore to aptamers (Figure 1B). A DNA aptamer was developed against pancreatic ductal adenocarcinoma (PDAC) by Cell SELEX [23] and labelled with Cy5. It has been applied on clinical PDAC tissue samples, along with a PDAC tumor-bearing mice model for in vivo imaging and detection of tumor [23]. Later, it was found to be specific against CD71, a human transferrin receptor, by mass spectrometry analysis [24]. As CD71 is overexpressed in PDAC along with other malignancies, this could be a potential diagnostic method for tumor biomarker detection.

#### 2.2.2. Colorimetric Detection

**Figure 2:**
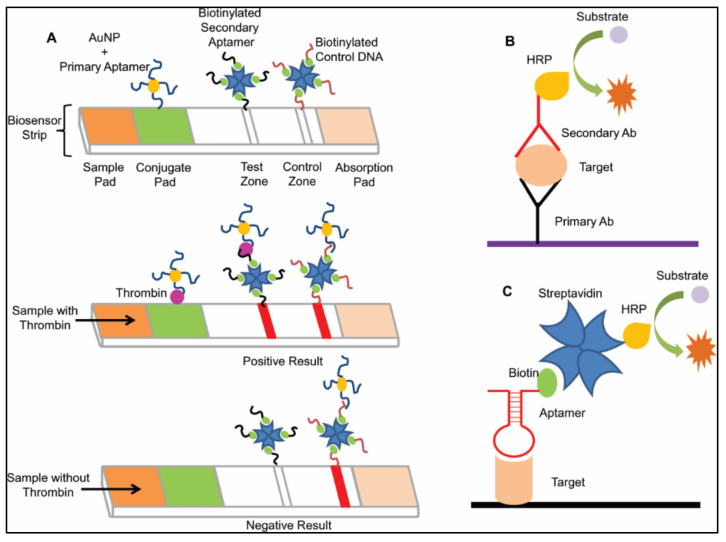
Schematic representation of aptamer-mediated diagnostic assays. (**A**) Lateral Flow Assay (LFA): Here, an aptamer-based strip sensor was used. After sample loading, the target molecule binds with the AuNP-conjugated primary aptamer. As the sample migrates, the target–aptamer–AuNP conjugate binds with a biotinylated secondary aptamer. Finally, the red band is shown at the test zone due to AuNP accumulation. In the control zone, the AuNP-primary aptamer binds with the control DNA to give the second red band. When the target molecule is absent, only the second red band is generated. (**B**) Conventional ELISA: Here, an antibody (Ab) is used to detect the target (**C**) ALISA: Here, an aptamer is used for target detection in place of an antibody.

The aptamer is affixed onto a gold nano particle (AuNP). Chemical (salt or cationic detergent)-mediated aggregation of AuNP finally help in target detection by producing visible color. Single-stranded oligonucleotides coat AuNP with their exposed nitrogenous bases, which, in turn, inhibit AuNP aggregation [25]. In the presence of a proper target, the ssDNA aptamer binds to it, leaving AuNP to aggregate and change color (Figure 1C). One such cationic detergent, Cetrimonium bromide (CTAB), mediated AuNP aggregation and helped in the detection of the Bisphenol A (BPA) level in different daily used products where a BPA-specific aptamer played a role [26]. This helped to regulate its level to below toxicity. A Point of Care Test (POCT) or bed-side test is a diagnostic test which is performed near the patient [27], among which the Lateral Flow Assay (LFA) is considered to be very popular. It is rapid, cost effective, easy to handle and has no requirement of trained personnel. The conventional LFA biosensor requires a target-specific antibody, but an aptamer can be a good choice of replacement to overcome disadvantages associated with an antibody [28]. For thrombin analysis, Liu et al. devised a biosensor strip with the aid of aptamers and AuNPs, which had superior sensitivity compared to a commercially available antibody-based strip sensor [29]. Capillary action helped the sample solution to migrate through the strip after being applied onto the sample pad. When it reached the conjugation pad, the target molecule was recognized by the primary aptamer coated onto AuNP. In the test zone, a biotinylated secondary aptamer, which was designed against a different epitope of same thrombin molecule, was immobilized by streptavidin. When the AuNP–primary aptamer–target complex was captured by a secondary aptamer, detection was possible by color production in the test zone due to AuNP accumulation, as a positive result. In the control zone, a complementary DNA probe, named control DNA, was affixed using the same principle, which was bound with an excess of AuNP–primary aptamer to develop a second colored band, which confirmed proper functionality of the biosensor strip. In the absence of thrombin, the second band was generated only at the control line [29] (Figure 2A). Besides POCT, like a rapid detection test (RDT), ELISA (Figure 2B) is one of the best plate-based protein detection methods in the laboratory, where the antibody is used as a detector, but the same approach can be achieved by an aptamer, called Aptamer Linked Immunosorbent Assay (ALISA) [25] (Figure 2C). Zeng et al. designed two different set ups: one with two different aptamers (2 and 10) against the Zika NS1 protein, as capturing and detection agents, respectively, and another with aptamer 2 as a capturing agent and an anti-NS1 antibody as a detection agent [30]. The hybrid aptamer–antibody assay was superior to the aptamer–aptamer assay due to its low limit of detection (0.1–1 ng/mL). It has shown its efficiency, even in a human serum sample, where proteases, other proteins and ions are present (detection limit >10 ng/mL), which indicates its future application in clinical detection. ALISA has been successfully used in detection of different forms of tuberculosis, like tuberculous meningitis [31], pleural tuberculosis [32], pulmonary tuberculosis [33], etc., with equal or even better sensitivity than available diagnostic techniques, like ELISA, microscopy smear or chest X ray.

#### 2.2.3. Pathogen Detection

Aptamers have been extensively used in infectious agent detection. For example, Wang et al. screened a DNA aptamer where a recombinant protein Hemagglutinin (HA) of H5N1 was used for the first few rounds (four cycles), followed by an entire virus particle as a target [34]. This mixed mode of selection helped in overcoming the drawbacks of aptamers that were raised against non-native recombinant proteins by using the aptamers to bind against the native protein. Aptamer-aided electrochemical sensors have also been used efficiently for pathogen detection. In packaged food and water, *E. coli* contamination was detected at as low as 10 cells/mL using aptamer-nanozyme technology on an electrochemical platform. Sharma et al. exploited the inherent peroxidase property of gold nanoparticles, which, when present on a bare surface, oxidized TMB, turning its color into blue. An *E. coli*-specific aptamer turned off the reaction by coating the AuNP surface in the absence of the pathogen, and the reaction was turned on by an aptamer–pathogen interaction [35]. Finally, H2SO4-mediated quenching of this reaction was detected with an electrochemical probe, and the captured electrical signal was found to be proportional to the bacterial load. This application could challenge available detection methods by its ease of handling, sensitivity and cost-effectiveness. The same principle was also applied by this group to detect other pathogens [36]. In a separate study, an electrochemical sensor was designed using a methylene blue (MB)-conjugated aptamer to detect tuberculosis meningitis in patient samples, where the electron transfer property of MB was used to design the probe, and the signal was turned off upon aptamer–antigen binding [37]. 

Some commercially available aptamer-based diagnostic products are listed in Table 1.

### 2.3. Therapeutics

In therapeutics, the aptamer can be used either as a carrier for therapeutic agents or as an inhibitory molecule itself. An aptamer conjugated with a liposome or nanoparticle helps in targeted drug delivery. QD, AuNP, GO, hydrogels, etc., act as vehicles, where aptamer coating helps in tissue targeting. 

#### 2.3.1. Therapeutic Drug Cargo

**Figure 3:**
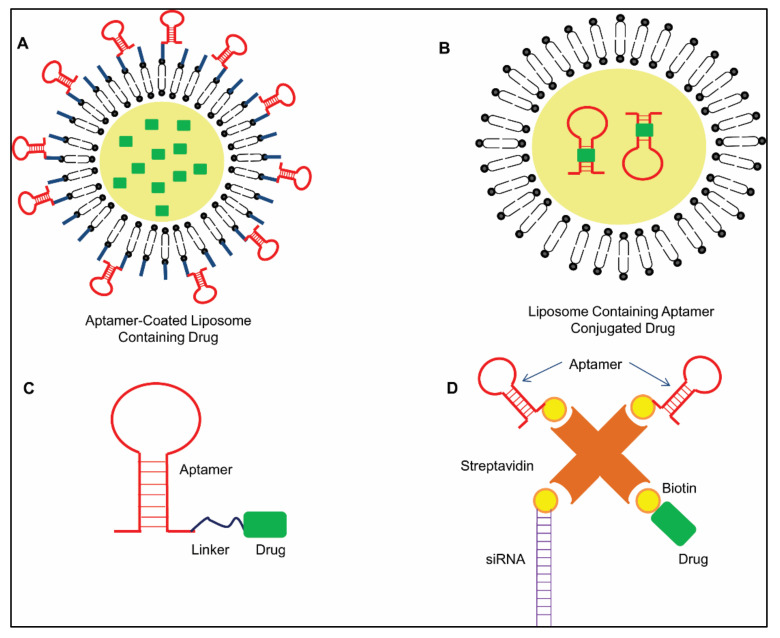
Schematic representation of the role of an aptamer in therapeutics. (**A**) Aptamer-coated liposome: A drug-containing liposome is conjugated with a cell surface marker-specific aptamer, which helps in targeted delivery of the drug. (**B**) Liposome containing an aptamer–drug conjugate: A drug like Doxorubicin is intercalated with an aptamer, and a drug–aptamer conjugate is coated by a cationic liposome, which enhances the delivery of the drug. (**C**) Linker-joined aptamer–drug conjugate: Short linkers are used to join the drug with an aptamer by click reaction. (**D**) Aptamer–siRNA–drug chimera: Streptavidin has four binding domains where the biotinylated aptamer, drug and siRNA bind. The aptamer helps in the targeted delivery of siRNA and the drug, whereas siRNA and the drug help in gene silencing and therapeutics at the desired target.

An aptamer against a tumor biomarker, named prostate-specific membrane antigen (PSMA), was affixed on a 2D structure of GO. This 2D structure was converted into a 3D structure with the aid of PEG to make a sieve where circulating tumor cells (CTC) of prostate cancer were being trapped due to the presence of a PSMA-specific aptamer. Although this construct was employed to detect CTC in blood [45,46], similar approaches could be performed for targeted drug delivery for therapeutic purposes. Liposome, an artificial, small, lipid bilayer vesicle, has been efficiently used for a long time as a cargo for a drug delivery system. Huwyler et al. designed monoclonal antibody-conjugated liposomes to encapsulate and deliver daunomycin, labelled with radioisotope tritium, directly to target cells [47]. However, several investigators replaced this antibody coating approach by aptamers. By click reaction, a PEGylated liposome was engineered in such a way to carry a cancer stem cell (CSC) marker CD44-specific aptamer onto it. This aptamer–liposome construct acted as an efficient drug delivery system for CD44 positive cells [48]. The same approach was previously used by Kang et al. for drug delivery to the leukemia cell line CEM-CCRF by using a sgc8-conjugated PEG functionalized liposome [49] (Figure 3A). Conversely, Plourde et al. exploited the property of nucleic acids to bind with intercalating drugs, like Doxorubicin (Dox) [50]. Encapsulation of the drug–aptamer complex into the cationic liposome (Figure 3B) with the aid of electrostatic interaction showed increased Dox incorporation into liposomes than the negative control. They also showed that in case of taubromycin, a lipophilic drug which usually has a low capacity of loading onto a liposome, an aptamer helped to overcome this drawback and finally increased the loading capacity by up to six times. The tumor-targeted delivery of a nanomaterial-encapsulated chemotherapeutic drug was also achieved by an aptamer. A PEG-functionalized biocompatible nanomaterial was used to encapsulate Docetaxel, which has previously shown potential cytotoxic effects in prostate cancer. Its efficacy was enhanced by coupling a PSMA-specific aptamer with a drug–nanoparticle bioconjugate by carbodiimide chemistry [51]. This RNA aptamer helped in the uptake of this bioconjugate, specifically by PSMA-expressing prostate epithelial cells. Therapeutic agents could also be directly guided to their specific target, either by intercalation with an aptamer or by complex formation via short linkers (Figure 3C). For example, Dox was linked to the sgc8c-aptamer by a short linker. The linker was connected to the aptamer and Dox via a click reaction and hydrazone moiety, respectively [52]. Unconjugated Dox was found to be more toxic to non-targeted cells than the aptamer–Dox conjugate, as the latter specifically killed the target cells only.

#### 2.3.2. Multiagent Cargo

Besides drugs, aptamers have been used as carriers for other nucleic acids, like miRNA, siRNA and DNAzyme, which act as gene regulatory elements. One such example is the aptamer–siRNA chimera (Figure 3D), where the efficacy of siRNA is improved, and targeted delivery is also achieved [53]. To construct an aptamer–siRNA chimera, a ribonucleic acid aptamer, A10, was connected to two different siRNAs against two genes, i.e., polo-like kinase 1 (PLK1) and B-cell lymphoma 2 (BCL2) [53]. These constructs targeted PSMA-overexpressed tumors, where an aptamer bound with PSMA and siRNA helped in silencing their respective genes, leading to a high therapeutic impact. Jeong et al. took an almost similar approach to overcome multidrug resistance of a breast cancer cell line, for which they targeted the underlying anti-apoptotic pathway by BCL2 gene silencing [54]. A polyvalent aptamer–doxorubicin–siRNA chimera was designed to target multidrug resistant MCF7 (MDR-MCF7) with an anti-Mucin 1 aptamer and BCL2-specific siRNA. Silencing the BCL2-mediated anti-apoptotic pathway triggered the sensitivity of tumor cells towards the drug Doxorubicin. This combinatorial pharmacogenomic approach of silencing the anti-apoptotic pathway and Doxorubicin release finally helped to accelerate the mortality of cancer cells via activating the apoptotic caspase-3/7 pathway. An RNA nanostructure, with a three-way junction (3WJ) or a four-way junction (4WJ) conformation, has been studied as a nanocarrier for drugs and other nucleic acids to treat aggressive cancer, where aptamers against cancer biomarkers have been used for their efficient delivery to the tumor site [55,56,57]. These structures are stable, non-immunogenic and efficiently carry therapeutic agents to the target site. An anti-CD133 aptamer was used as an extended arm of the 3WJ RNA nanostructure to target triple-negative breast cancer (TNBC), which efficiently delivered anti-miR21 to inhibit the oncogenic nature of miR21 [55]. In another study, a 4WJ RNA nanobody was constructed, where an anti-EGFR aptamer was incorporated as one of the four arms [57]. Each of the arms was capable of carrying six molecules of paclitaxel, and a total of 24 molecules of the drug were delivered by each nanostructure (4WJ-X-24-PTXs) to the TNBC tumor site with the help of an anti-EGFR aptamer. This technology not only helped in targeted delivery, but it also reduced the toxicity of Paclitaxel. 

#### 2.3.3. Inhibitory Agent

An aptamer itself can act as an inhibitor by binding and inhibiting the interaction of its target protein with the downstream partners, and thus, it can impede signaling pathways. Yang et al. demonstrated an aptamer molecule against the envelop protein E1E2 of the Hepatitis C virus [58]. This aptamer molecule was found to inhibit viral infection. In another study, it was reported that a Spiegelmer against CXCL12, named NOX-A12, inhibited the interaction between a C-X-C chemokine receptor type 4 (CXCR4) and its ligand, C-X-C motif chemokine 12 (CXCL12) [59]. Clinical trials are ongoing to assess the role of NOX-A12 (Olaptesed pegol) in different aspects of pancreatic and colorectal cancers, such as increasing the infiltration rate of immune cells as well as sensitivity of tumors to checkpoint inhibitors, along with inhibition of tumor repair mechanisms. The CXCR4–CXCL12 interaction triggers tumor cell growth, migration and invasion. Hence, this inhibitory aptamer undoubtedly has a great potential as a drug molecule [59].

#### 2.3.4. Immune Modulators

Cancer immunotherapy has become popular as an alternative approach to chemo- and radio-therapy, where immune checkpoint proteins are targeted with specific antibodies. However, antibody-mediated immunotherapy has been facing certain challenges, such as a monoclonal antibody-mediated auto-reactive response, expensive treatment, manufactural difficulty, bioengineering issues, etc., [60], which, in turn, have led to the development of oligonucleotide aptamers as alternate immune modulators. Several immune checkpoint aptamers have been developed and studied in vitro and in vivo, and their role in cancer immunotherapy has been explored [60,61]. First, an immune checkpoint aptamer has been reported against murine CTLA4 [62]. A CTLA4 blockade led to T cell expansion and enhanced anti-tumor T cell activity. The tetrameric structure of this anti-CTLA4 aptamer increased avidity, circulation time and efficacy in vivo. The inhibition of the Programmed cell death protein 1–Programmed cell death ligand 1 (PD1–PDL1) interaction is another important pathway for targeted therapy. Prodeus et al. screened the oligonucleotide library to select an anti-PD1 aptamer, MP7, which selectively blocked the PD1–PDL1 interaction and suppressed IL2-mediated immune suppression [63]. To enhance its poor pharmacokinetic profile, 40 PEG molecules were added and injected to an MC38 syngenic mice model. This aptamer showed an equivalent efficiency to an anti-PD1 antibody in suppressing the growth of PDL1+ colon cancer cells, if not lesser. Similarly, an aptamer against PDL1 has been identified by the nitrocellulose filter SELEX [64]. This 45-nucleotide long aptamer could recognize both murine and human PDL1 and modulate the tumor microenvironment by elevated CD4+ and CD8+ T cell infiltration and IL2, IFNγ, TNFα and other chemokine expression at the tumor site, which might form a loop against tumor proliferation. Besides antagonists, aptamers have played the role of agonists to produce co-stimulatory signals and, thus, tumor regression. A multimeric aptamer against 4-1BB was studied to generate a co-stimulatory signal and recruit CD8+ T cells after T cell receptor (TCR) activation [65]. A monomeric aptamer was not able to elicit stimulation, whereas a divalent aptamer was found to generate antitumor immunity in vivo. This might be due to the requirement of receptor crosslinking to turn on signaling cascades. The same concept worked behind an anti-OX40 aptamer, where Dollins et al. used a DNA scaffold to construct an oligomeric RNA aptamer, which, in turn, acted as an OX40 agonist and stimulated the anti-cancer activity of T cells [66].

#### 2.3.5. Aptamers in Clinical Trials

Macugen (Pegaptanib sodium) was approved as a drug after getting clearance from the U.S. Food and Drug Administration (FDA) in the year of 2004 to treat patients suffering from age-related macular degradation (AMD). Pegaptanib was developed for anti-VEGF therapy, as VEGF plays a crucial role in ocular neovascularization [67]. Human VEGFA contains several isoforms due to alternative splicing, among which VEGF165 is predominant [68]. VEGF165 carries both of the heparin binding (HBD) and receptor binding (RBD) domains, in which HBD is exclusive for VEGF165 and does not present in other isoforms, including VEGF121 [68,69]. Macugen was developed against VEGF 165 with several modifications, like PEGylation and base modifications, to improve its stability and circulation time in body fluids [70]. Although it could not bind with the RBD region, it inhibited angiogenesis by binding with HBD and impeding receptor binding to RBD through some steric hindrance or inhibiting the interaction of VEGF 165 with Proteoglycan, Heparan sulphate or Neuropilin1 [69]. In spite of its early success, Macugen failed to become the most promising candidate for treating AMD, as it could not inhibit the VEGF–VEGFR interaction by RBD and, thus, VEGF-mediated angiogenesis. The mAb-based drug Lucentis has now become preferred over Macugen, as it can bind with RBD of all the isoforms and have a broad range of target recognition [71], but an aptamer against the same target domain might be able to acquire an equal potential. Thus, Nonaka et al. designed an aptamer, V7t1, exclusively against RBD, which was able to interact with both the isoforms VEGF165 and VEGF121 [72]. They screened this aptamer in order to increase the affinity of detection by constructing a bivalent aptamer using HBD-specific del5_1 [73] and RBD-specific V7t1 [72]. The potential role of the V7t1 aptamer as a substitute of Lucentis should be explored and requires extensive study.

Currently, multiple aptamers are in phase I, phase II and phase III clinical trials and waiting for approval, some of which are listed in Table 2.

## 3. Chemical Modifications of Aptamer

**Figure 4:**
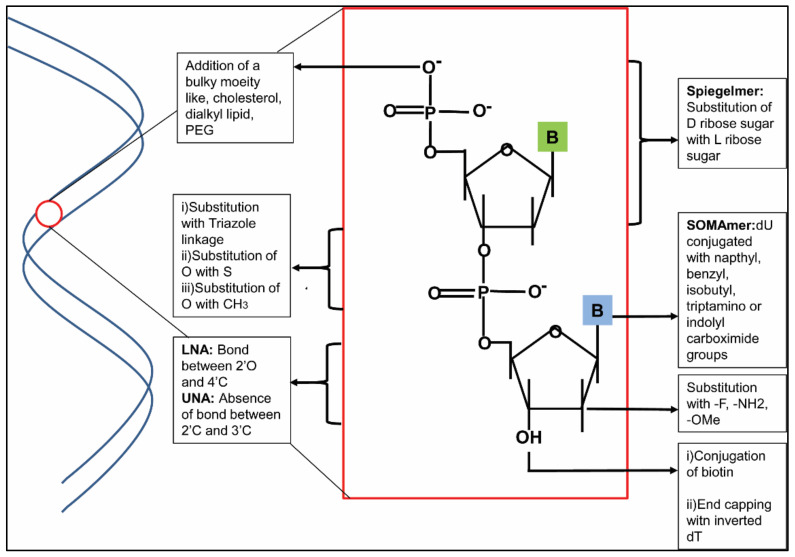
Schematic representation of different modifications in the aptamer. Modifications can be done at the 5′ and 3′ ends of the sugar phosphate backbone, by substitution of phosphodiester linkage, by using modified bases or by changing the configuration of sugar.

Inside biological system aptamers encounter several hindrances to be effective as diagnostic and therapeutic tools. Having a molecular weight of 6–30 kDa, they are highly prone to renal clearance [100,101]. Thus, their circulation time in biological fluid should be increased via modifications in such a way that they can exceed the filtration limit (50 kDa). Being oligonucleotides, these aptamers are also prone to nuclease degradation [100,101]. Unmodified aptamers can be stable in body fluid for a maximum of up to several minutes, which can also be countered by modifications. Modifications can be done at the 5′ and/or 3′ end of the sugar phosphate backbone of aptamers, at the nucleotides of aptamers or at the linker (Figure 4). 

### 3.1. End Modifications of the Backbone

The sugar phosphate backbone of the aptamer molecule can be modified at the 5′ and/or 3′ end: (i) 3′ modifications: Inverted thymidine-mediated 3′ end capping (Figure 4) is effective to increase aptamer stability, as well as to inhibit 3′ exonuclease degradation in human serum. Several aptamers, which are in clinical trials, like Pegaptanib, ARC1779 and BAX499 modified with 3′ inverted thymidine, have shown an improved half-life [102]. The 3′ biotin conjugation (Figure 4) also provides resistance against nuclease degradation and increases its circulation time in the blood. Dougan et al. found that a biotin-conjugated aptamer against thrombin improved circulation time as well as nuclease resistance in vitro, whereas the biotin–streptavidin conjugated aptamer had improved half-life in blood circulation as well [103]. (ii) 5′ modifications: To increase the aptamer stability in body fluid or to save it from renal clearance, some bulky moieties are added at the 5′ end, such as cholesterol, a dialkyl lipid, PEG molecule, etc., (Figure 4). Smidt et al. modified the 5′ end of an oligonucleotide by conjugating a cholesterol moiety via a phosphate spacer to make a 16 mer cholesteryl oligonucleotide (cholODN). In plasma, this cholODN had a longer half-life (9–11 min) than the unmodified one (<1 min) [104]. 

### 3.2. Modifications of Sugar

Sugar is modified by changing the bonds, adding a bulky moiety or using an enantiomer: (i) Locked Nucleic acid (LNA) indicates a bond between 2′O and 4′C (Figure 4), which is highly resistant to nuclease degradation. Mallikaratchy et al. modified an aptamer against mIgM BCR (TD05) with LNA [105]. They substituted purines and pyrimidines of stem by LNA and found that pyrimidine substitution increased the affinity as well as stability against nuclease attack, but purine substitution in the stem region impaired binding. Unlocked Nucleic Acid (UNA) refers to the absence of a bond between 2′C and 3′C (Figure 4), which provides thermostability as well as flexibility to the aptamer. When a 15 mer thrombin aptamer was modified in its loop region with UNA, its thermostability was increased, but the same modification in the G quartet region hampered G quadruplex formation [106]. (ii) 2′ modifications: 2′ fluorine, amine or O-methyl modifications also serve the same functions [107] (Figure 4). (iii) Mirror Image DNA: The idea of Spiegelmer or Mirror image DNA emerged in 1996 [108,109]. These are basically composed of an L-ribose sugar, which is an enantiomer of normal D ribose sugar (Figure 4). Being L ribose, these Spiegelmers are highly resistant to nuclease degradation. However, these enantiomeric DNAs cannot bind with normal protein, which is composed of L amino acid. Rather, this L form of DNA binds with D amino acid, which is an enantiomer of L amino acid. First, this protein sequence is chemically synthesized, which comprises of the D form of the corresponding amino acids. Then, the normal D aptamer is selected against this D protein. The L aptamer of the selected D aptamer is synthesized, which is then able to bind a normal L protein of the system. Klussmann et al. has generated a 58 mer L-RNA aptamer against D adenosine, which had a 9000-fold higher affinity towards D adenosine than L adenosine and nuclease resistance as well [108]. First, a bioactive Spiegelmer was synthesized by Bartel et al. against the nona peptide hormone Vasopressin (VP). They synthesized an L aptamer against VP, which has shown activity in a cultured kidney cell by antagonizing the activity of its target [110]. Later, Spiegelmers against various protein targets were synthesized [111], some of which among them are in clinical trials. One such promising Spiegelmer is NOX A12 against CXCL12, which has already been discussed briefly in this review.

### 3.3. Modifications of Phosphodiester Linkage

(i) Triazole linkage replacement at the position of phosphodiester linkage (Figure 4) is another promising approach against nuclease digestion, which can be achieved either by an automated method, using a dinucleoside block for phosphoramidite synthesis, or via a click reaction between modified nucleosides bearing alkyne and azide groups [112]. (ii) Phosphodiester linkage is also modified by phosphorothioate (the substitution of O with –S) and methylphosphonate (the substitution of O with –CH3) (Figure 4) [112]. The substitution of O with S provides a negative charge to the phosphate backbone, which, in turn, makes it more stable. Substitutions of both nonbridging O atoms with S atoms generate Phosphorodithioate linkage, which is even more stable than the earlier one [112]. 

### 3.4. Modifications of the Base

To increase target diversity, functional groups, which can mimic the structure of amino acid side chains, are conjugated, which, in turn, provide them with a protein-like property, as well as diverse secondary and tertiary structures. They can interact with more epitopes of the target and have a slow dissociation rate (slow off-rate) [113]. These are recognized as Slow Off-rate Modified Aptamers, or SOMAmers (Figure 4), with better binding affinity, along with binding kinetics and nuclease resistance activity, over traditional unmodified aptamers. The modification of a guanine quadruplex anti-nucleolin aptamer, AS1411, with 5-(N-benzylcarboxyamide)-2-deoxyuridine (5-BzdU) has been found to be effective in increasing its binding affinity to cancer cells. This benzyl group can also be replaced by napthyl, triptamino, isobutyl, etc., which also help to increase the affinity [113].

## 4. Role of Aptamer in COVID-19 Control

SARS-CoV-2 is a positive strand RNA virus, which has almost 79.5% sequence similarity with SARS-CoV of coronaviridiea family. It has a non-structural gene containing two overlapping ORFs, which, in turn, are spliced and coded into proteins required for viral replication, four structural genes coding for Spike (S), Membrane (M), Envelope (E) and Nucleocapsid (N) proteins and some other accessory protein-coding genes. Spike protein is the main culprit against viral infection. It has two domains: S1 recognizes its receptor, ACE2, on epithelial cells, after which S2 helps in the internalization into host cells [114,115]. This transmembrane spike protein can be a potent candidate for aptamer–target recognition. Not only the viral protein, but also its RNA can be used for detection to bypass the RT-PCR or CRISPR Cas-mediated detection system. This review will cover a spectrum of different approaches of aptamer-mediated viral detection, along with its potential role for COVID-19 treatment in this current situation.

### 4.1. Detection of Nucleic Acid

Viral RNA detection in patient samples has been considered as the most reliable method of confirmation until now. Fluorogenic RNA is the best candidate for this approach. MS2, SPINACH and MANGO are some of these fluorogenic RNAs, among which MANGO performs best in terms of its low background signal and high binding affinity. MANGO is an RNA aptamer which has a conserved loop to bind with thiazole orange-based fluorescent dye (TOI), and it fluoresces brightly upon binding [116]. This fluorogenic aptamer can be coupled with nested Nucleic Acid Sequence Based Amplification (NASBA) technology to get the desired result in real time. Like nested PCR, two sets of primers have also been here designed against target RNA, between which the inner primer sequences carry the MANGO aptamer sequence, which, in turn, folds and binds when the corresponding fluorophore is provided and gives the RNA level in the sample. One of the advantages of this technology is the usage of only three enzymes (T7 RNA Pol, Rnase H and Reverse transcriptase) and two sets of primers [117,118].

Unrau et al. developed this method and detected clpB protein in *E. coli* at an attomolar level (1.5 RNA molecules/microliter) [118]. This sensitive method could be applied in the current COVID-19 scenario to detect viral RNA directly from patient samples in a shorter time than the conventional RT PCR. Primers designed against a unique sequence of SARS-CoV-2 and a minimal region of the MANGO aptamer sequence will be incorporated. Multiplex Nested Mango NASBA (NMN) can also be applied, where the outer primers will be same, but inner primers can vary according to different mutations of different SARS-CoV-2 strains or can nullify false negatives by amplifying different regions of the same viral genome to increase the stringency [118]. 

### 4.2. Detection of Protein

The surface protein of SARS-CoV-2 can be considered as the best candidate in this regard. As the Spike protein is the interacting partner between the virus and human cell, an aptamer designed against the S protein may have a potential outcome in rapid detection. Several types of aptasensors have already been characterized and applied in viral detection.

After the breakout of SARS-CoV, Oh et al. devised an aptamer-based chemiluminescence assay for the detection of the SARS-CoV nucleocapsid protein [119]. This sandwich assay, with a capturing aptamer and detecting antibody, also provided a comparable detection limit (2 pg/mL) with an available ELISA. Le et al. devised a Dual Recognition Element Lateral Flow Assay (DRELFA) for the detection of the influenza virus, where the antibody was paired with an aptamer to overcome their limitations of cross-reactivity and slow-binding kinetics, respectively [120]. Their DRELFA successfully differentiated between different strains of the influenza virus with a better sensitivity compared to an antibody-based existing diagnostic kit. Fluorescence can be detected by the change of its intensity or polarization. The molecular beacon is one of the well-exploited tools to devise a fluorescence aptasensor. Kumar et al. devised a split aptamer technique to detect the HIV Tat protein, where they derived two oligomers from a Tat protein-binding aptamer RNA^Tat^ [121]. One of the oligomers (F-BA1-D) of this split aptamer contained both fluorescence (Fluorescein) and quencher (DABCYL) molecules, which formed a hairpin structure and quenched fluorescence in the absence of a target protein. In the presence of a target, the second unstructured oligomer (DA2) bound with the former to attain the proper. RNA^Tat^ conformation, which, in turn, interacted with the target, and the fluorescence got recovered. The above-mentioned approaches may help researchers to discover future insights into COVID-19 detection, bypassing the antibody-based immunoassays with a similar or more sensitive and less costly aptamer-based detection technique. One or two aptamers with a high specificity along with a high affinity against the viral surface protein could suffice.

### 4.3. Therapeutics for COVID-19

Literature studies have shown different therapeutic applications of aptamers as direct candidates or cargo partners in various viral diseases. Researchers targeted different proteins involved in viral replication to inhibit infection with the help of target-specific aptamers. A modified RNA aptamer worked efficiently against the methyl transferase enzyme of dengue virus and inhibited its replication and translation [122]. RNA-dependent RNA polymerase (RdRP) is important for RNA virus replication and, thus, infection. The inhibitory effect of an aptamer against RdRP has already been investigated in HCV infection [123]. Being an RNA virus, targeting RdRP and other non-structural proteins involved in viral replication could be a potential treatment for SARS-CoV-2 infection as well. Zhou et al. investigated the dual role of an aptamer–siRNA chimera against HIV infection, where the aptamer against the gp120 envelop protein rendered a gp120–CD4 interaction as well as helped in target-specific internalization of siRNA [124].

This siRNA exerted a therapeutic effect against infection by silencing Tat/Rev protein expression.

### 4.4. Recent Advancements

After the outbreak of COVID 19, researchers from different countries have been in a constant search of an affordable, quick and easy to use detection kit, and an aptamer has been one of their prime choices. This review will discuss some of the recent advancements in diagnostic and therapeutic pipelines.

Prof. Dr. Mayer and his group have developed the aptamer SP6 against the spike protein of SARS-CoV-2, which neither interacted with the RBD domain nor inhibited the viral interaction with its receptor, ACE2, but significantly reduced the viral load [125]. Although the mechanism is still unknown, it has been speculated to be post-interaction, i.e., the prevention of spike protein cleavage or the destabilization of prefusion conformation of the same. This finding has opened up a new insight into RBD-independent COVID-19 detection and treatment which may play a crucial role when RBD undergoes mutations. Liu et al. were the first group to synthesize aptamers against the nucleocapsid protein that plays a crucial role in the structure and transmission of SARS-CoV-2 [126]. Four high-affinity aptamers were used for target detection with the aid of an antibody in both LFA and gold nanoparticle immunochromatographic strip (GIS) platforms. An antibody–aptamer mixed approach was able to detect its target at the 1 ng/mL level. As these aptamers interacted with different regions of the N protein, a sandwich assay with a better detection efficiency is the key advantage of this study.

Like other countries, India is also putting efforts into designing a cost-effective quick diagnosis kit. Dr. Sharma’s group at Translational Health Science and Technology Institute (THSTI) has developed a panel of high affinity (low nanomolar) DNA aptamers against spike trimeric antigen of SARS-CoV-2. They have characterized spike binding aptamers using various biochemical and biophysical techniques. Structural analysis of best performing aptamer candidate (S14) evinced that it binds to the N-terminal domain of the protein but not to the receptor binding domain. They have also validated the performance of S14 aptamer in nasopharyngeal swab specimens (n=232). Aptamer-based assay developed by his group was able to detect SARS-CoV-2 infection with a sensitivity and specificity of ∼91 % and 98 %, respectively. Another important finding of this study was that they have tested 7 different batches of aptamers but did not see any batch-to-batch variation. This observation clearly demonstrates an important attribute of aptamers, invariability in performance regardless of batches [127]. Dr. Ramalingam from the Vellore Institute of Technology (VIT), with his teams, is in process of devising aptamer-based LFA detection kits separately for COVID-19 diagnosis, which promise prompt, easy and sensitive POC testing [128].

The outbreak of SARS-CoV-2 is the current ongoing concern due to its pandemic nature. From Wuhan, China, it has now spread all over the world, across 210 countries. A total of 167,492,769 people have affected to date (26 May 2021) from late December, 2019, with 3,482,907 deaths reported by World Health Organization (WHO) [129]. This pandemic situation necessitates quick, cheap and efficient diagnosis for early quarantine and treatment. An aptamer could be the future weapon of choice in our battle against COVID-19.

## 5. Future Prospect: Aptamers in Stem Cell Research, Single Cell Proteomics and Immunotherapy

**Figure 5:**
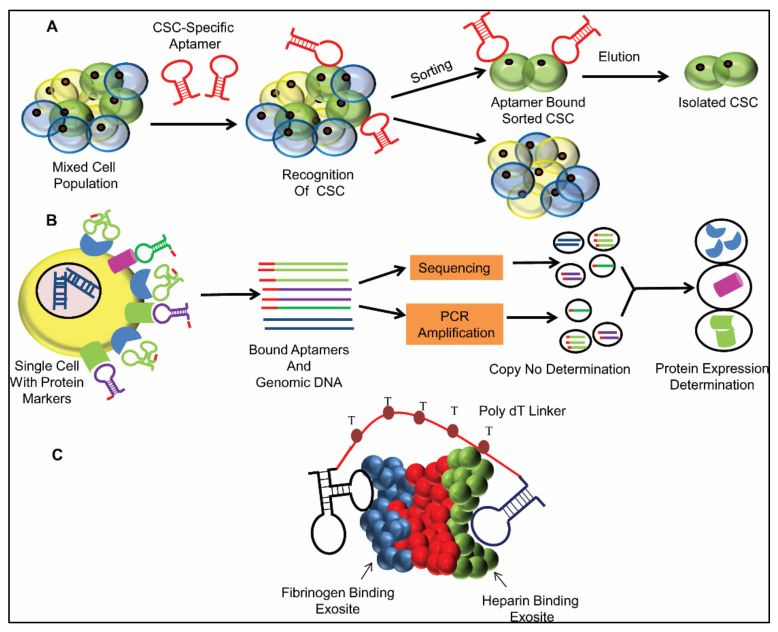
Schematic representation of the Future applications of aptamers: (**A**) Aptamer-mediated cancer stem cell isolation: In the tumor site, there is a mixed cell population. The CSC-specific aptamer can only bind to the particular CSC marker against which it is generated. Aptamer-bound CSCs are distinguished from the heterogeneous population. (**B**) Role of aptamers in Single Cell Proteomics: Unique sequence-tagged aptamers bind with different protein targets present on a single cell. Targeted Single cell sequencing determines the copy number of each of the bound aptamers, which, in turn, provide the expression level of the corresponding proteins or the nature of scProteomics. (**C**) Aptamer dimerization via a linker: Two different aptamers bind with a thrombin protein at two different domains, i.e., the fibrinogen-binding site (blue) and heparin-binding site (green), respectively, and are connected by various lengths of a poly dT linker, among which the five-thymine linker is the most suitable one.

Stem cells contain some unique features or markers, contrasted against differentiated cells. Aptamers against these markers are being used in stem cell recognition in a mixed population, stem cell isolation, stem cell targeted therapy and tracking or imaging stem cell differentiation. Iwagawa et al. developed three aptamers (L1-65, L2-2 and L3-3) against mouse embryonic stem cells [130]. The targets of these aptamers were expected to be expressed in the transitional state of differentiation but not in fully differentiated cell lines (NB2a, A9, C2C12). These were expected to be helpful in tracking embryonic stem cell differentiation into ectodermal and mesodermal cells. An interesting work by Yoon et al. showed that the CD31-specific aptamer could isolate an endothelial progenitor cell from a mixed population, which, in turn, when transplanted to a murine hind limb ischemia mice model, could restore blood flow [131]. Tumor-initiating cells (TICs) or cancer stem cells (CSCs) are the main culprit behind therapy resistance, cancer recurrence, metastatic spread, etc. The identification of these cells itself is a challenge. Kim et al. developed aptamers against Glioblastoma (GBM) TICs by the cell SELEX approach, where they used TICs for positive selection and human neural progenitor cells (NPCs) for negative selection, respectively [132]. These TICs-specific aptamers may have a potential for several applications, like drug therapy for GBM, imaging of the tumor site, exploring the mechanism and subtypes of GBM, etc. Besides this, the EpCAM-specific aptamer has been reported to have been used to isolate EpCAM-positive cells from a mixed population of cells [133]. Several aptamers, available against CSC markers (CD44, CD133, ABCG2, etc.), can be used as a bait to trap cancer stem cells from a heterogeneous population (Figure 5A).

Single cell proteomics is basically the snapshot of the protein landscape of a single cell for a given time [134]. Single cell omics is a powerful depiction of tumor heterogeneity, among which scProteomics data is the most reliable one, as it shows the actual translational status of each and every cell. However, available technologies (Flow cytometry, Mass cytometry, etc.) do have several pitfalls, like poor sensitivity, inefficient sampling procedure, reduced multiplexing capacity, etc., [134]. The analysis of a maximum of 50–100 proteins in a single go has been achieved to date, which is actually a very minute part of the entire proteome [135]. This drawback can be overcome using nucleotide aptamers. Aptamers can be conjugated with different heavy metal ions and used in Mass CyTOF in place of an antibody, which could make the technique cost-effective. Beyond this, hundreds of thousands of aptamers, bound to a single cell with their respective protein targets, can be screened simultaneously using Targeted Sequencing. These aptamers should be tagged with a common unique sequence that must not be present within the human genome. This unique sequence can act as an adaptor and be captured by a common complementary sequence. Single Cell Targeted Sequencing can help to sequence only these captured aptamer molecules, rather than the whole genome, which would reduce the time as well as the cost. This approach can finally provide the copy numbers of different protein-bound aptamers, which, in turn, provides the final status of the expression level of corresponding proteins, i.e., proteomics data with single-cell resolution (Figure 5B). 

One of the recent advancements in immunotherapy is the application of a bispecific antibody (bsAb), i.e., a monoclonal antibody (mAb) designed against two different antigens [136,137]. Two different heavy and light chain variable regions, specific for two different proteins or antigens, are engineered together into a single molecule, in order to construct a bsAb, which can bind both of the corresponding targets, present in the same cell (cis interaction) or in different cells (trans interaction), simultaneously or sequentially [137]. This crosstalk helps in a synergistic outcome by recruiting effector molecules or cells. The advantage of a bispecific or multispecific antibody is the low level of toxicity compared to the simultaneous use of their parent monoclonal counterparts [136]. This idea can lead to a new direction in aptamer therapeutics. Bispecific or multispecific aptamers, synthesized chemically using different available linkers, can be a better choice over the complicated in vivo synthesis of bsAb. Linker type and length should be optimized such that proper binding of the desired cis or trans mode can be achieved without any steric hindrance. These linkers play an important role in aptamer multimerization and, hence, in increasing affinity as well as avidity [138]. The role of a bivalent/multivalent aptamer has already been investigated by several researchers. Hasegawa et al. synthesized a bivalent aptamer by connecting a 15 mer aptamer against the fibrinogen-binding region and a 29 mer aptamer against the heparin-binding region of a thrombin protein with varying lengths (1 to 20 mer) of the poly dT stretch, and they found the 5-mer linker to be the most suitable to provide the required flexibility [73] (Figure 5C). Another group, Mallikaratchy et al. found that the affinity increment of aptamers became independent of linker length beyond a certain point while working with bivalent and multivalent aptamers against a B cell receptor (BCR) [105]. Therefore, the length of a spacer molecule is very important in the case of multidentate aptamers concatenation, and it should be flexible to fit perfectly with the distance between the different epitopes of the target protein [138]. This may have promising implementations in different human diseases, including malignancies where aptamers against different target proteins act together in the activation or/and inhibition of their respective binding partners to alter different signaling pathways. The synthesis, binding property, affinity, mode of action and therapeutic applications of multispecific/multivalent aptamers demand extensive study that would explore a new horizon in the near future.

Interpatient and intratumor heterogeneity is a common reason for the failure of prevailing chemotherapy. Thus, precision medicine is currently crucial for the success of cancer treatment. The aptamers identified by SELEX (via a biomolecule and/or Cell-based) against heterogeneous targets or a heterogeneous population of cancer cells are anticipated to strengthen the efficacy of existing cancer therapies with reduced side effects.

## 6. Conclusions

Over past few decades, aptamer technology has been recognized as an alternative molecular tool for detection and treatment. From small molecules to toxins and from proteins to whole organisms, aptamers have been developed and used for the detection of almost all types of targets. Hence, aptamers are being used as detector molecules in various industries, as well as in medical fields. Several diagnostic kits have already been developed with promising industrial and clinical applications. Similarly, aptamers play a crucial role as therapeutic candidates. With their wide range of applications, ranging from inhibitory molecules to drug cargo to immune modulators, aptamers outcompete antibodies in several directions. Many of them are already in clinical trials, having a potential role in human health. Together, these approaches help to develop the oligonucleotide aptamer as a strong theranostic candidate. This theranostic property, in turn, enlightens future anticipation in biomedical applications and draws the attention of researchers for extensive study and investigation. 

## Figures and Tables

**Table 1:** Aptamer-based products in the diagnostic pipeline.

Product Name	Developer	Applications	Detection Mode	References
Aptocyto	Aptamer Science Inc.	Isolation of biomarker positive cells from a heterogeneous population using a magnetic bead	Flowcytometry-based detection	[38]
Aptoprep	Aptamer Science Inc.	Aptamer-based protein (biomarker) pull down from sample using magnetic beads	Fluorescence-based detection	[38]
AflaSense	Neoventerus Biotechnology Inc.	Fungal aflatoxin detection in food industry	Fluorescence-based detection	[38]
CibusDx	CibusDx	Food-borne and water-borne pathogen detection	Electrochemical-based detection	[39]
OLIGOBIND	Sekisui Diagnostics	Active Thrombin level detection in plasma sample	Fluorogenic activity-based detection	[40]
OTASense	Neoventerus Biotechnology Inc.	Fungal Ochratoxin A detection in food industry	Fluorescence-based detection	[38]
SOMAscan	SomaLogic	Novel biomarker detection associated with different diseases	SOMAmer-based detection	[41,42,43,44]

**Table 2:** Aptamers in Different Phases of Clinical Trials.

Aptamer	Target	Medical Condition	Clinical Status	References	Trial Identifier (Clinical Trials.gov Identifier)
ARC1779	von Willebrand factor	von Willebrand’s disease	Phase III (Awaiting)	[74,75,76]	NCT00432770 NCT00507338 NCT00632242 NCT00694785 NCT00726544 NCT00742612
ARC1905	Complement Factor 5 (C5)	Neovascular age-related macular degeneration	Phase I	[77]	NCT00709527 NCT00950638 NCT02686658 NCT03362190 NCT03364153 NCT03374670
ARC19499	Tissue Factor Pathway Inhibitor (TFPI)	Haemophilia	Terminated	[78]	NCT01191372
AS1411	Nucleolin	Advanced solid tumors, Renal cell carcinoma, Acute myeloid leukaemia (AML)	Phase III (Awaiting)	[79,80]	NCT00512083 NCT00740441 NCT00881244 NCT01034410
E10030	PDGF	Von Hippel Lindau disease, Age-related macular degeneration	Phase III (Awaiting)	[81,82]	NCT00569140 NCT01089517 NCT01940887 NCT01940900 NCT01944839 NCT02591914 NCT02214628 NCT02859441
EYE001	VEGF	Wet age-related macular degeneration	Phase I (Completed)	[83,84]	NCT00021736 NCT00040313 NCT00056199 NCT00150202 NCT00239928 NCT00321997 NCT00736307
NOX-A12	CXCL12	Chronic lymphocytic Leukaemia, Multiple myelomas, Metastatic pancreatic and colorectal cancer	Phase II	[85,86]	NCT00976378 NCT01194934 NCT01486797 NCT01521533 NCT01947712 NCT03168139
NOX-E36	CCL2	Type II diabetes mellitus	Phase II	[87,88]	NCT00976729 NCT01085292 NCT01372124 NCT01547897
NOX-H94	Hepcidin	Anaemia of chronic inflammation	Phase I (Completed)	[89]	NCT01372137 NCT01522794 NCT01691040 NCT02079896
NU172	Thrombin	Coronary artery disease	Phase II	[90]	NCT00808964
Pegaptanib (Macugen)	VEGF165	Age-related Macular Degeneration (AMD), Diabetic macular oedema, Uveitis, Diabetic cystoid oedema, Proliferative Diabetic Retinopathy (PDR)	In market	[70,91,92]	NCT00406107 NCT00549055 NCT00788177 NCT00790803 NCT01175070 NCT01486771 NCT01487070 NCT01573572
RB006	Factor IX	Coronary artery disease	Phase III (Awaiting)	[93,94]	NCT00715455 NCT00932100 NCT01872572
REG1 System	Factor IX	Coronary artery disease, Acute coronary syndrome	Phase III (Terminated)	[95,96,97]	NCT00113997 NCT00715455 NCT00932100 NCT01848106 NCT02435082 NCT02797535 etc.
Sgc8	PTK7	Colorectal cancer	Recruiting	[98,99]	NCT03385148

## Data Availability

Not applicable.
